# Multiblock Analysis to Relate Polyphenol Targeted Mass Spectrometry and Sensory Properties of Chocolates and Cocoa Beans

**DOI:** 10.3390/metabo10080311

**Published:** 2020-07-29

**Authors:** Noémie Fayeulle, Sébastien Preys, Jean-Michel Roger, Renaud Boulanger, Clotilde Hue, Véronique Cheynier, Nicolas Sommerer

**Affiliations:** 1 SPO, INRAE, Univ Montpellier, Institut Agro—Montpellier Supagro, 34060 Montpellier, France; noemie.fayeulle@laposte.net (N.F.); veronique.cheynier@inrae.fr (V.C.); nicolas.sommerer@inrae.fr (N.S.); 2 Ondalys, 34830 Clapiers, France; 3 ITAP, INRAE, Univ Montpellier, Institut Agro—Montpellier Supagro, 34060 Montpellier, France; jean-michel.roger@inrae.fr; 4 ChemHouse Research Group, 34060 Montpellier, France; 5 CIRAD, UMR Qualisud, 34398 Montpellier, France; renaud.boulanger@cirad.fr; 6 Qualisud, Université de Montpellier, CIRAD, Montpellier SupAgro, Université d’Avignon, Université de La Réunion, F-34000 Montpellier, France; 7 Valrhona SA, 26600 Tain l’Hermitage, France; clotildehue33@gmail.com

**Keywords:** polyphenols, mass spectrometry, sensory, chocolate, chemometrics, multiblock analysis

## Abstract

Chocolate quality is largely due to the presence of polyphenols and especially of flavan-3-ols and their derivatives that contribute to bitterness and astringency. The aim of the present work was to assess the potential of a quantitative polyphenol targeted metabolomics analysis based on mass spectrometry for relating cocoa bean polyphenol composition corresponding chocolate polyphenol composition and sensory properties. One-hundred cocoa bean samples were transformed to chocolates using a standard process, and the latter were attributed to four different groups by sensory analysis. Polyphenols were analyzed by an ultra-high-performance liquid chromatography (UPLC) system hyphenated to a triple quadrupole mass spectrometer. A multiblock method called a Common Component and Specific Weights Analysis (CCSWA) was used to study relationships between the three datasets, i.e., cocoa polyphenols, chocolate polyphenols and sensory profiles. The CCSWA multiblock method coupling sensory and chocolate polyphenols differentiated the four sensory poles. It showed that polyphenolic and sensory data both contained information enabling the sensory poles’ separation, even if they can be also complementary. A large amount of variance in the cocoa bean and corresponding chocolate polyphenols has been linked. The cocoa bean phenolic composition turned out to be a major factor in explaining the sensory pole separation.

## 1. Introduction

Chocolate is the result of the complex transformation of the seeds of *Theobroma cacao* called cocoa beans. A wide range of molecules causes the flavor perceived while tasting dark chocolate. Volatile compounds [1,2,3,4], including pyrazines [5,6,7,8], and nonvolatile compounds such as fatty acids [9] and polyphenols are directly involved in the chocolate flavor (taste and odor). Both the composition of the beans and the different parameters applied along the chocolate-making process have an influence on the chocolate flavor [10,11,12,13,14]. Polyphenols are present in large quantities in cocoa beans compared to other plant materials [15,16,17,18,19] and key molecules for the cocoa bean and chocolate quality. Thus, cocoa is one of the major dietary sources of polyphenols and especially of flavan-3-ols, i.e., catechin monomers and their oligomers and polymers, called procyanidins [20,21]. These molecules are appreciated for their potential health beneficial effects [15,16,17,22,23] but also known to be bitter and astringent [24,25,26]. They have therefore an impact on the chocolate taste [27,28,29,30,31] and can even be markers for fine flavor cocoa beans [32,33].

High-performance liquid chromatography (HPLC) or ultra-high-performance liquid chromatography (UPLC) coupled with mass spectrometry (MS) is the most commonly used method to analyze natural products [34] and, in particular, phenolic compounds [35]. Untargeted MS analysis has been successfully applied to establish polyphenol fingerprints of cocoa extracts [36] and, with the aid of MS/MS fragmentation, enabled the characterization of previously unknown compounds [16,36,37,38,39]. However, a targeted analysis using a triple quadrupole (QqQ) instrument operating in the multiple reaction monitoring (MRM) detection mode, providing higher selectivity and sensitivity, is usually preferred for quantitative analysis [35].

Only a few studies have explored the relationships between sensory analysis and cocoa or chocolate polyphenol compositions [25,28,29,40]. Taste characteristics and perception thresholds of isolated phenolic compounds have been determined using half-tongue tests [27,29]. Taste omission experiments performed in a water solution confirmed the identification of taste active compounds [28], but this approach does not take into account eventual interactions with other chocolate components. Statistical tools have been especially developed to relate sensory data with chemical data [41] and successfully applied to other food such as wine [42,43] but have seldom been tried on chocolate or cocoa data [36]. Among them, the Common Component and Specific Weights Analysis (CCSWA) is a chemometrics multiblock method that enables the description of the relationships between several data matrices dealing with the same samples and having different numbers of variables [41].

The objective of this study is to understand the link between a sensory analysis of chocolate samples and the polyphenol contents of chocolate and cocoa samples using a multiblock approach. Therefore, the first part is dedicated to the analysis of the link between the sensory data and chocolate polyphenol composition, and the second part analyzes the link between the cocoa and chocolate polyphenol contents, determined by UPLC-QqQ-MS in the MRM mode, since polyphenols are expected to change along the chocolate-making process.

## 2. Results and Discussion

### 2.1. Differentiation of Chocolate Samples in the Sensory Space

First, a PCA (principal component analysis) was performed on the sensory data to assess the differences between the four sensory poles in the sample set provided by our industrial partner.

**Figure 1:**
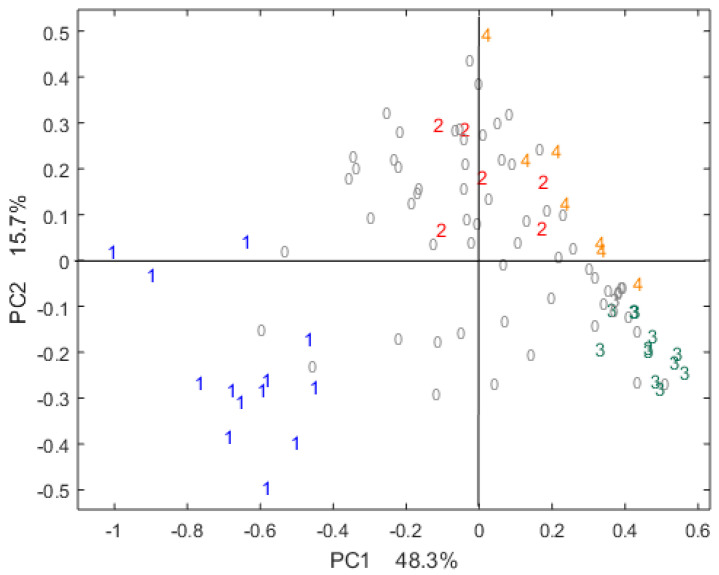
Projection of the samples on the first two scores of the principle component analysis (PCA) performed on centered sensory data. Sensory pole cores are coded from 1 to 4 with different colors: 1 = core pole 1, 2 = core pole 2, 3 = core pole 3, 4 = core pole 4 and 0 = samples not belonging to any core pole.

Figure 1 shows the scores of the PCA on the sensory data for the first two components, representing 48.3% and 15.7% of the total variance, respectively. These first results show the cocoa bean diversity among our samples, which is typical for the chocolate industry [44]. This diversity is essential to build robust models. Samples from the same sensory pole core are well-grouped. This is confirmed when using the third component as well (not shown).

### 2.2. Identification of the Polyphenolic Compounds in the Chocolate Samples Linked to the Sensory Analysis

**Figure 2:**
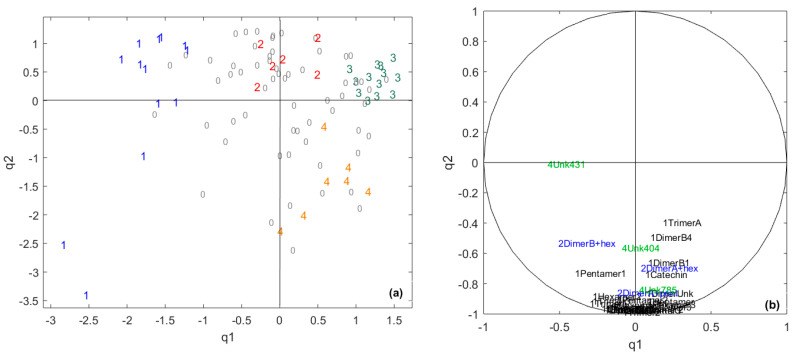
(**a**) Projection of the samples on the two first scores of the Common Component and Specific Weights Analysis (CCSWA) on sensory and chocolate polyphenols data for the two first common components, the so-called “common space”. 1 = core pole 1, 2 = core pole 2, 3 = core pole 3, 4 = core pole 4 and 0 = samples not belonging to any core pole. (**b**) The correlation circle for the polyphenol variables. Variables are coded with different colors: black = flavan-3-ols, blue = glycosylated flavan-3-ols and green = unknown compounds.

A CCSWA (Common Component and Specific Weights Analysis) was performed on the two data blocks corresponding to chocolate polyphenol composition and sensory scores. The first common dimension represents 4% of the total variance for the polyphenol data and 48% for the sensory data (Table 1). The second one represents 8% of the total variance of the sensory data and 70% of the polyphenol data. As shown in Table 1, the so-called “common space” between the two datasets can be found in the plane q1–q2 (Figure 2). The common space is studied first to detect the information common to both the sensory data and polyphenol data.

The common percentage of the variance, i.e., specific weights associated to this “common space” is low when considering component-by-component, suggesting that the link between chocolate polyphenols and sensory data is rather weak. The value obtained for the RV coefficient is 21%, confirming the weak link between the two tables. This seems logical, as the sensory poles are mainly differentiated by aroma characteristics, which come from volatile compounds [1,11,45], while polyphenols are sapid compounds [28]. However, a better differentiation between the sensory pole cores was observed using both sensory and chocolate polyphenol datasets than using only the sensory dataset (Figure 2a).

The polyphenol variables involved in the sensory pole discrimination appear to be mainly flavan-3-ols, which are known to contribute astringency and bitterness to cocoa [28].

**Figure 3:**
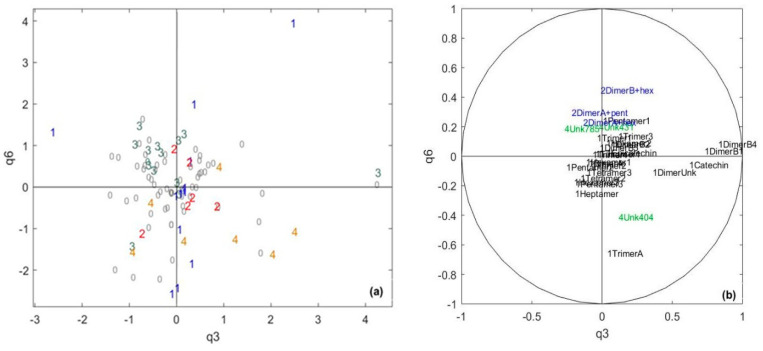
The CCSWA on the chocolate and sensory data, the specific space of the polyphenolic chocolate data: (**a**) score plot and (**b**) correlation circle. 1 = core pole 1, 2 = core pole 2, 3 = core pole 3, 4 = core pole 4, and 0 = samples not belonging to any core pole. Black = flavan-3-ols, blue = glycosylated flavan-3-ols and green = unknown compounds.

Then, the specific dimensions of the polyphenolic data were studied on the q3-q6 plane, highlighting the information about polyphenols that is not related to the sensory data (Figure 3). No sensory pole core separation was achieved. Component q6 seems to be positively built with variables corresponding to glycosylated procyanidin dimers and negatively built with higher molecular weight flavan-3-ols. Glycosylated flavan-3-ols arise from the hydrolysis of sucrose into glucose and fructose, which react with flavan-3-ols such as procyanidin dimers to form C-glycosides, as described earlier [29]. Indeed, these reactions can be promoted by heating in the cocoa fermentation or chocolate-processing steps. Flavanol C-glycosides have been reported to exhibit astringency, with human recognition thresholds comprised between 1 and 95 µm/L, depending on their structure, when tasted by the half-tongue test [29]. However, the present data (the fact that these variables do not contribute to the space common to both the sensory data and polyphenol data) suggest that these molecules have limited impacts on chocolate sensory perceptions. Component q3 is mainly built on catechin, dimer B1 and dimer B4, which both contain catechin units. These compounds are in very low quantities compared to other flavan-3-ols isomers derived from epicatechin in chocolate [46,47]. Therefore, they are less likely to be involved in sensory discrimination, as they hardly can be perceived during the chocolate tasting [27,28]. Moreover, the correlation between catechin derivatives and a lack of correlation with epicatechin derivatives suggests different formation mechanisms. This might reflect differences related to their biosynthesis in cocoa (due to genetics and/or environmental factors) or the epimerization of epicatechin to catechin promoted by high temperatures [48,49]. This is in agreement with earlier studies showing, using a chiral analysis, that the levels of (–)-epicatechin were related to those of (+)-catechin, the natural epimer, but not those of (¬)-catechin, resulting from the epimerization of (–)-epicatechin, which is the major form in chocolate.

### 2.3. Exploring the Link between Cocoa and Chocolate Polyphenols Contents

Another CCSWA was performed on the cocoa polyphenol and chocolate polyphenol data to describe the link between the two data blocks.

A RV coefficient of 70% was obtained, illustrating the close relationship between these two datasets. The specific weights associated to each component show that there is a “common space” on components q1 and q4, where q1 represents 50% and 68% of the variance of cocoa beans and chocolate polyphenols, respectively, and q4 represents 3% and 5% of the variance of cocoa beans and chocolate polyphenols, respectively (Table 2). These common components will provide information on the common behaviors of polyphenols along the chocolate-making process.

**Figure 4:**
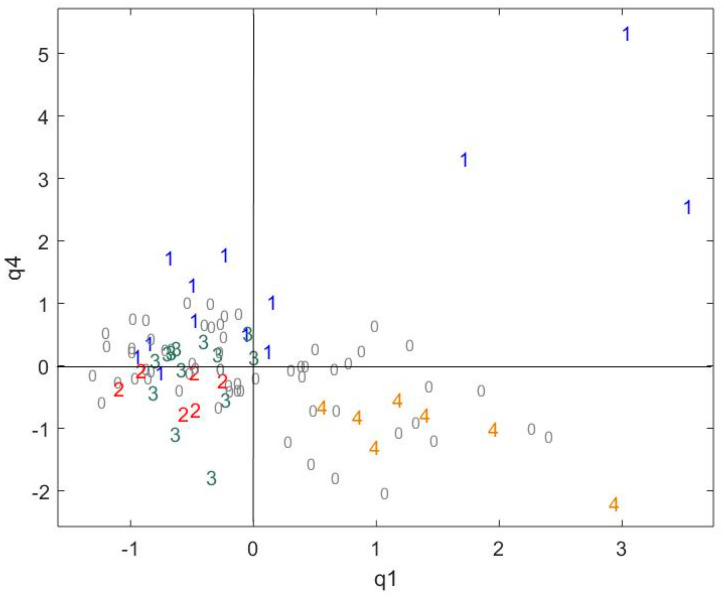
The CCSWA on the chocolate and cocoa data, a sample projection in the common space q1–q4. 1 = core pole 1, 2 = core pole 2, 3 = core pole 3, 4 = core pole 4 and 0 = samples not belonging to any core pole.

The sample projection shows that there is a trend in the sensory core pole separation (Figure 4). Core poles 1 and 4 are well-separated from each other and from poles 2 and 3, which overlap. This confirms that the polyphenol data is partly related to the sensory core pole separation.

**Figure 5:**
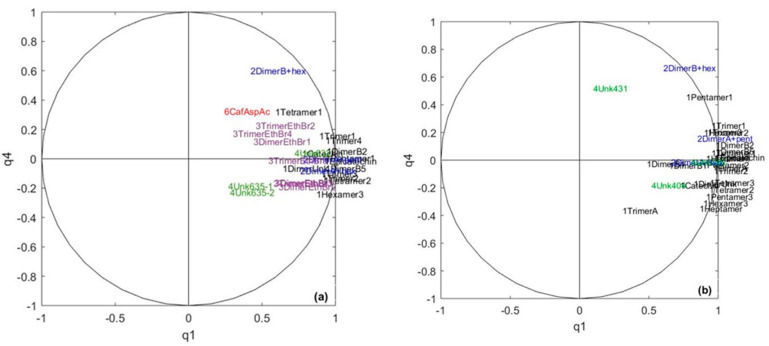
The CCSWA on the chocolate and cocoa data: (**a**) correlation circle in the common space for cocoa variables and (**b**) correlation circle in the common space for chocolate variables. Black = flavan-3-ols, blue = glycosylated flavan-3-ols, green = unknown compounds, red = hydroxycinnamic acid and purple = ethyl bridged flavan-3-ols.

Given the variable organization and the percentage of explained variance, it seems that the first component q1 reflects the global concentration of polyphenols both in cocoa and in chocolate (Figure 5a,b). Even if the quantity of flavan-3-ols drastically drops along the chocolate-making process [48,49], there are still flavan-3-ols in chocolate [49,50,51] in quantities proportional to those present in cocoa beans. A B-type dimer linked to a hexose for both cocoa and chocolate and an unknown molecule for chocolate are the main variables building q4. As mentioned earlier, flavan-3-ol glycosides may result from a reaction of flavan-3-ols with glucose and fructose released by sucrose hydrolysis, which is also affected by fermentation (e.g., invertase enzyme activity released by some microorganisms and a temperature increase induced by fermentation). This result confirms what was obtained in the previous CCSWA on the specific dimensions of chocolate polyphenols (Figure 3).

**Figure 6:**
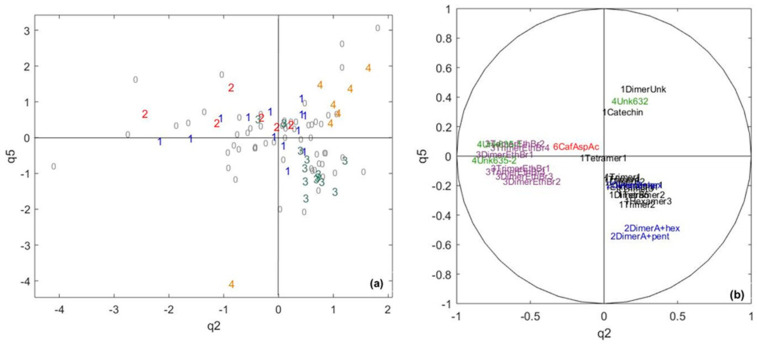
The CCSWA on the chocolate and cocoa data, a cocoa-specific space: (**a**) score plot. 1 = core pole 1, 2 = core pole 2, 3 = core pole 3, 4 = core pole 4 and 0 = samples not belonging to any core pole. (**b**) Correlation circle. Black = flavan-3-ols, blue = glycosylated flavan-3-ols, purple = ethyl-bridged flavan-3-ols, green = unknown compounds and red = hydroxycinnamic acid.

Studying the specific components for cocoa polyphenols data highlights specific information for cocoa polyphenols that is not related to chocolate polyphenols. Components q2 and q5 appeared suitable to represent specific components of cocoa polyphenols, as they represented 22% and 6% of the cocoa polyphenol variance for only 2% and 1.7% of the chocolate polyphenol variance (Figure 6). Pole 1, together with 2, 3 and 4, are quite well-separated, but one sample from pole 4 is completely apart from all the other samples.

Component q2 is mainly built by ethyl-bridged flavan-3-ols and two unknown polyphenol molecules and separating poles 1 and 2 from poles 3 and 4. Ethyl-bridged flavan-3-ols are molecules mainly formed during cocoa bean fermentation through the condensation of two flavan-3-ols with acetaldehyde produced by microorganisms [37]. This suggests that the quantity of ethyl-bridged flavan-3-ols in fermented cocoa is correlated with pole 1 and 2 sensory profiles. These molecules have been reported to contribute both astringency and bitterness [52]. In addition, they appear uncorrelated to flavan-3-ols, which seems logical given their formation pathway. However, the correlation may also reflect the composition of the fermentation microflora and its metabolism, known to be involved in the formation of flavor precursors that yield flavor compounds during roasting. The presence of flavan-3-ols in general, and especially of glycosylated flavan-3-ols, seems to differentiate poles 3 and 4, suggesting that a higher concentration of these molecules in fermented cocoa may be associated with pole 3. On the contrary, pole 4 seems to present a higher amount of catechin, an unknown procyanidin dimer, and an unknown compound. As explained above, a temperature elevation can cause the epimerization of the main flavan-3-ols (i.e., (–)-epicatechin, dimer B2 and dimer B5), leading to the formation of (–)-catechin and dimers containing (–)-catechin units, as shown earlier for procyanidin A2 [53]. Our data suggests that these reactions, which have been reported to occur under conditions mimicking conching, may also take place during fermentation. However, since the (–)-catechin cannot be distinguished from the natural (+)-catechin epimer under our analytical conditions, a further analysis considering each epimer separately is needed to confirm this hypothesis.

Components q3 and q7 illustrate the specific planes of chocolate polyphenols as they represent 7% and 3% of the chocolate variance, respectively, and only 1.5% and 0.4% of the cocoa variance (not shown). Component q3 is mainly built by flavan-3-ols based only on epicatechin, which are uncorrelated to catechin, and dimer B1, dimer B4 and the unknown dimer, which is consistent with the previous CCSWA on chocolate and sensory data. Component q7 is built on unknown nitrogenous compounds and glycosylated flavan-3-ols. However, as no sensory pole separation is achieved, no further conclusion can be made from this specific component.

## 3. Materials and Methods

### 3.1. Chocolate and Cocoa Samples

One-hundred fermented and dried cocoa beans and corresponding chocolate samples were provided by Valrhona SA, Tain l’Hermitage, France. For each sample, 10 kg of cocoa was transformed into chocolate by a standardized process. An aliquot of 100 g of the cocoa or chocolate sample was ground in liquid nitrogen to obtain a thin and homogenous powder. Polyphenols were extracted using the protocol described in our previous work [36]; each sample has been extracted three times in order to obtain biological replicates. Samples were stored at −20 °C before analysis.

### 3.2. Polyphenols Analysis by UPLC-ESI-TQ-MS

Polyphenols were analyzed by an Acquity UPLC system (Waters, Saint-Quentin-en-Yvelines, France) hyphenated to a triple quadrupole (QqQ) TQD mass spectrometer (Waters, Saint-Quentin-en-Yvelines, France). The UPLC system was composed of a binary pump and a cooled autosampler maintained at 7 °C and equipped with a 5-µL sample loop, a 100-µL syringe and a 30-µL needle and a diode array detector (DAD). MassLynx software (Waters, Milford, MA, USA) was used to control the instruments and to acquire the data, which was then processed with TargetLynx software (Waters, Milford, MA, USA). Chromatographic separation was achieved by a reversed-phase Acquity HSS T3 1.8-µm 1.0 × 100-mm column (Waters, Saint-Quentin-en-Yvelines, France) protected by a 0.2-µm in-line filter and maintained at 40 °C. The mobile phase consisted of 1% (*v*/*v*) formic acid in deionized water (solvent A) and 1% (*v*/*v*) formic acid in methanol (solvent B). The flow rate was 0.17 mL/min. Samples were injected into the column by using the Partial Loop with Needle Overfill injection mode with an injection volume of 1 µL. The elution program was a linear multistep gradient (time (min), %B): (0, 5), (8, 6), (10, 10), (13, 16), (17, 16), (22, 35), (25, 45), (26.5, 99), (29.5, 99), (30, 5) and (34, 5). The mass spectrometer was operated in MRM mode with electrospray ionization (ESI) in the negative ionization mode. The source and desolvation temperatures were respectively set at 120 °C and 450 °C. Nitrogen was used as the desolvation (500 L/h) and cone (50 L/h) gas. Argon was used as the collision gas at a flow rate of 0.16 mL/min. Capillary voltage was set at 3.5 kV in the positive mode and 2.8 kV in the negative mode.

Forty different compounds were investigated: 27 compounds were analyzed in the cocoa samples and 29 compounds in the chocolate samples. Some compounds were in both lists, while some were specific to each matrix (Table 3). Flavan-3-ols and their derivatives were the main targeted compounds, and seven compounds were unknown. These molecules are the most relevant variables for sensory pole discriminations selected by nontargeted mass spectrometry analysis and identified as described earlier [36].

MRM conditions have been optimized for each of them, and calibration curves were performed with the injection of known concentrations of standard compounds. Details are in Tables S1 and S2 (Supplementary Materials). Commercial standards of (+)-catechin, (–)-epicatechin and procyanidin dimers B1 and B2 were purchased from Extrasynthèse (Genay, France); procyanidin dimers B4 and B5 and procyanidin trimer C1 were purchased from PlantMetaChem (Gießen, Germany) and procyanidin dimer A2 and quercetin-3-O-glucoside were purchased from Sigma Aldrich (Saint-Louis, MI, USA). External calibration was performed with a mixture of those commercial standards. To assess the absence of suppression effects in the ionization process due to coelutions, we spiked a sample with the standards at different concentrations, and we did not note any effect due to the coelutions. Data is expressed in mg of compound per g of cocoa or chocolate sample and detailed in Table S3 (Supplementary Materials).

### 3.3. Sensory Analysis of Chocolate Samples

The chocolate samples were assigned to four sensory groups using a quantitative descriptive analysis (QDA) performed by an internal trained panel from our industrial partner Valrhona SA, as described earlier [1]. Each group represents a trend in chocolate flavor called a “sensory pole”. They are labeled poles 1 to 4. Each sensory pole is composed of both “typical” samples, which fit the sensory requirements well, and other samples, which represent the diversity encountered in plant matrices. The typical samples of each sensory pole build what we call “sensory pole cores”.

### 3.4. Data Analysis

Principal component analysis (PCA) was used to explore the sensory data in a multivariate way.

The multiblock analysis called Common Component and Specific Weights Analysis (CCSWA) was used to establish a link between chocolate polyphenols and sensory data and, then, between cocoa and chocolate polyphenols. The purpose of the CCSWA is to summarize the information of the different data matrices, also called blocks, by a set of vectors (scores or common components) q1, q2, … , qk representing the common underlying dimensions [41]. An originality of the CCSWA is to associate a specific weight with each block for each common component. When several blocks have close values of their weight for a given component, it is an indication that this dimension represents common information for these tables. Inversely, a higher weight value for one block compared to the others for a given dimension reflects a specific contribution of this block to the sample mapping observed on this dimension. The CCSWA will therefore find common and specific spaces relating to different blocks of data. In this paper, the CCSWA is used to analyze two blocks corresponding to two different analytical techniques for the same samples (sensory and polyphenols using MS on chocolates) and, then, to analyze two blocks corresponding to two process points for the same samples (cocoa beans and chocolates).

Polyphenol data were centered and column-wise standardized because of a large intensity variability, while sensory data was only centered.

The RV coefficient is calculated as the multivariate generalization of the determination coefficient R^2^ between 2 matrices [54,55].

All data treatments were achieved using MATLAB software (The MathWorks, Inc., Natick, MA, USA).

## 4. Conclusions

The present study aimed at exploring the relationships between chocolate sensory and polyphenol data and between cocoa and chocolate polyphenol compositions. The link between the chocolate polyphenols and sensory date is rather weak, consistent with the large impact of aroma compounds on sensory pole discrimination [1,2]. The CCSWA multiblock method coupling sensory data and chocolate polyphenol data differentiated the four sensory core poles, even in a better way than using sensory data alone, meaning that the two datasets contained relevant and complementary information. The main polyphenol variables involved in the sensory pole discrimination were flavan-3-ols and, especially, epicatechin derivatives. These molecules have been reported to contribute astringency and bitterness to cocoa [28]. Specific dimensions of the polyphenol data highlighted additional information. In particular, glycosylated flavan-3-ols and catechin derivatives, arising from the reactions of cocoa flavan-3-ols during chocolate processing, discriminated chocolate samples but were not related to the sensory data. Although these molecules contribute to astringency, they had a limited impact on chocolate sensory perception, presumably due to their low concentrations [27,28].

Another CCSWA analysis performed on cocoa and chocolate polyphenol data showed strong links between the two datasets, the concentration of polyphenols and, especially, flavan-3-ols in chocolate depending on their concentration in cocoa beans. Moreover, the cocoa bean composition turned out to be a major factor in explaining the sensory poles’ separation. Indeed, the quantity of some particular polyphenols (ethyl-bridged flavan-3-ols, glycosylated flavan-3-ols, catechin and dimer B1) in fermented and dried cocoa beans could be related to the sensory pole attribution. This suggests that, although these molecules are not present in sufficient quantities to affect the chocolate taste, they can serve as markers of botanical or geographical origins and, in particular, reflect the fermentation conditions and the metabolism of the microflora performing cocoa bean fermentation that affect the organoleptic properties of the final product.

## Figures and Tables

**Table 1:** Values of the specific weights (%) of each component (q1 through q9) calculated by the Common Component and Specific Weights Analysis (CCSWA) with chocolate polyphenolic data and sensory data.

Data Table/Common Component	q1	q2	q3	q4	q5	q6	q7	q8	q9
Sensory	0.48	0.08	0.00	0.15	0.06	0.00	0.00	0.04	0.00
Polyphenols in chocolates	0.04	0.70	0.07	0.00	0.00	0.04	0.03	0.00	0.01

**Table 2:** Values of the specific weights (%) of each component calculated by the CCSWA with the chocolate polyphenolic data and cocoa polyphenolic data.

Specific Weights	q1	q2	q3	q4	q5	q6	q7	q8	q9
Polyphenols in cocoa beans	0.50	0.22	0.01	0.03	0.06	0.03	0.00	0.02	0.02
Polyphenols in chocolates	0.68	0.02	0.07	0.05	0.01	0.01	0.03	0.00	0.00

**Table 3:** List of the different polyphenol variables analyzed in this study.

Codes	Molecules Studied	Presence	Molecule Family
Catechin	Catechin	Cocoa + chocolate	Flavan-3-ol
Epicatechin	Epicatechin	Cocoa + chocolate	Flavan-3-ol
DimerB1	Procyanidin dimerB1	Chocolate	Flavan-3-ol
DimerB2	Procyanidin dimer B2	Cocoa + chocolate	Flavan-3-ol
DimerB4	Procyanidin dimer B4	Chocolate	Flavan-3-ol
DimerB5	Procyanidin dimer B5	Cocoa + chocolate	Flavan-3-ol
DimerUnk	Unknown procyanidin dimer	Cocoa + chocolate	Flavan-3-ol
A-type Dimer	Procyanidin A type dimer	Chocolate	Flavan-3-ol
Trimer1	Procyanidin trimer	Cocoa + chocolate	Flavan-3-ol
Trimer2	Procyanidin trimer C1	Cocoa + chocolate	Flavan-3-ol
Trimer3	Procyanidin trimer	Cocoa + chocolate	Flavan-3-ol
Trimer4	Procyanidin trimer	Cocoa + chocolate	Flavan-3-ol
TrimerA	A type procyanidin trimer	Chocolate	Flavan-3-ol
Tetramer1	Procyanidin tetramer	Cocoa + chocolate	Flavan-3-ol
Tetramer2	Procyanidin tetramer	Cocoa + chocolate	Flavan-3-ol
Tetramer3	Procyanidin tetramer	Chocolate	Flavan-3-ol
Pentamer1	Procyanidin pentamer	Cocoa + chocolate	Flavan-3-ol
Pentamer2	Procyanidin pentamer	Chocolate	Flavan-3-ol
Pentamer3	Procyanidin pentamer	Chocolate	Flavan-3-ol
Hexamer1	Procyanidin hexamer	Chocolate	Flavan-3-ol
Hexamer2	Procyanidin hexamer	Chocolate	Flavan-3-ol
Hexamer3	Procyanidin hexamer	Cocoa + chocolate	Flavan-3-ol
Heptamer	Procyanidin heptamer	Chocolate	Flavan-3-ol
DimerB+hex	B type procyanidin dimer C-hexoside	Cocoa + chocolate	Glycosylated flavan-3-ol
DimerA+hex	A type procyanidin dimer O-hexoside	Cocoa + chocolate	Glycosylated flavan-3-ol
DimerA+pent	A type procyanidin dimer O-pentoside	Cocoa + chocolate	Glycosylated flavan-3-ol
TrimerEthBr1	Ethyl Bridged procyanidin trimer	Cocoa	Ethyl bridged flavan-3-ol
TrimerEthBr2	Ethyl Bridged procyanidin trimer	Cocoa	Ethyl bridged flavan-3-ol
TrimerEthBr3	Ethyl Bridged procyanidin trimer	Cocoa	Ethyl bridged flavan-3-ol
TrimerEthBr4	Ethyl Bridged procyanidin trimer	Cocoa	Ethyl bridged flavan-3-ol
DimerEthBr1	Ethyl Bridged procyanidin dimer	Cocoa	Ethyl bridged flavan-3-ol
DimerEthBr2	Ethyl Bridged procyanidin dimer	Cocoa	Ethyl bridged flavan-3-ol
DimerEthBr3	Ethyl Bridged procyanidin dimer	Cocoa	Ethyl bridged flavan-3-ol
CafAspAc	Caffeoyl aspartic acid	Cocoa	Hydroxycinnamic acid
Unk632	Unknown compound at m/z 632	Cocoa	Unknown
Unk635-1	Unknown compound at m/z 635	Cocoa	Unknown
Unk635-2	Unknown compound at m/z 635	Cocoa	Unknown
Unk404	Unknown compound at m/z 404	Chocolate	Unknown
Unk431	Unknown compound at m/z 431	Chocolate	Unknown
Unk785	Unknown compound at m/z 785	Chocolate	Unknown

## Supplementary Materials

The following are available online at https://www.mdpi.com/2218-1989/10/8/311/s1: Table S1: Multiple reaction monitoring (MRM) acquisition parameters for cocoa extracts in the negative ion mode. Table S2: Multiple reaction monitoring (MRM) acquisition parameters for chocolate extracts in the negative ion mode. Table S3: Cocoa and chocolate polyphenols’ experimental data.
