# Supplementary material for: Multiblock Analysis to Relate Polyphenol Targeted Mass Spectrometry and Sensory Properties of Chocolates and Cocoa Beans
Source: Metabolites. 2020 Jul 29;10(8):311. doi: 10.3390/metabo10080311 (PMC7465875; doi:10.3390/metabo10080311)
Supplement: Supplementary file 1 [file metabolites-10-00311-s001.zip › supplementary/Submission special issue Metabolites_Supplementary file S1_200527.docx]

**Table S1.** Multiple Reaction Monitoring (MRM) acquisition parameters for cocoa extracts in the negative ion mode.

| **Compound Code** | **Molécule** | **Precur-sor Ion (m/z)** | **Reten-tion Time (min)** | **Cone Volta-ge (V)** | **Qualif. ion (m/z)** | **Coll. Energy (V)** | **Quantif. ion (m/z)** | **Coll.  Energy (V)** | **Quantified as equivalent of** |
| --- | --- | --- | --- | --- | --- | --- | --- | --- | --- |
| **Catechin** | Catechin | 289 | 6.1 | 35 | 205 | 15 | 245 | 15 | Catechin |
| **Epicatechin** | Epicatechin | 289 | 12.3 | 35 | 205 | 15 | 245 | 15 | Epicatechin |
| **DimerB2** | Procyanidin dimer B2 | 577 | 11.5 | 35 | 289 | 25 | 425 | 15 | Dimer B2 |
| **DimerB5** | Procyanidin dimer B5 | 577 | 20.5 | 35 | 289 | 25 | 425 | 15 | Dimer B5 |
| **DimerUnk** | Unknown Procyanidin dimer | 577 | 13.9 | 35 | 289 | 25 | 425 | 15 | Dimer B1 |
| **Trimer1** | Procyanidin trimer | 865 | 10.3 | 50 | 125 | 45 | 287 | 30 | Dimer B2 |
| **Trimer2** | Procyanidin trimer C1 | 865 | 14.3 | 50 | 125 | 45 | 287 | 30 | Dimer B2 |
| **Trimer3** | Procyanidin trimer | 865 | 15.4 | 50 | 125 | 45 | 287 | 30 | Dimer B2 |
| **Trimer4** | Procyanidin trimer | 865 | 21.4 | 40 | 125 | 50 | 287 | 30 | Dimer B2 |
| **Tetramer1** | Procyanidin tetramer | 576^(2-)^ | 12.4 | 25 | 125 | 30 | 289 | 15 | Dimer B2 |
| **Tetramer2** | Procyanidin tetramer | 1153 | 15 | 50 | 289 | 60 | 407 | 60 | Dimer B2 |
| **Pentamer1** | Procyanidin pentamer | 720^(2-)^ | 14.1 | 30 | 125 | 35 | 289 | 20 | Dimer B2 |
| **Hexamer3** | Procyanidin hexamer | 864^(2-)^ | 17.8 | 35 | 125 | 50 | 289 | 45 | Dimer B2 |
| **DimerB+hexose** | B-type dimer C-hexoside | 739 | 10.7 | 35 | 619 | 20 | 449 | 15 | Dimer B2 |
| **DimerA+hexose** | A-type dimer O-hexoside | 737 | 19.9 | 40 | 449 | 30 | 611 | 25 | Dimer A2 |
| **DimerA+pentose** | A-type dimer O-pentoside | 707 | 20.3 | 50 | 449 | 30 | 581 | 20 | Dimer A2 |
| **TrimerEthylBridge1** | Ethyl Bridged procyanidin trimer | 893 | 22.8 | 40 | 451 | 30 | 603 | 20 | Epicatechin |
| **TrimerEthylBridge2** | Ethyl Bridged procyanidin trimer | 893 | 24.2 | 40 | 451 | 30 | 603 | 20 | Epicatechin |
| **TrimerEthylBridge3** | Ethyl Bridged procyanidin trimer | 893 | 25.7 | 40 | 451 | 30 | 603 | 20 | Epicatechin |
| **TrimerEthylBridge4** | Ethyl Bridged procyanidin trimer | 893 | 26.1 | 40 | 451 | 30 | 603 | 20 | Epicatechin |
| **DimerEthylBridge1** | Ethyl Bridged procyanidin dimer | 605 | 24.4 | 30 | 289 | 20 | 315 | 20 | Epicatechin |
| **DimerEthylBridge2** | Ethyl Bridged procyanidin dimer | 605 | 25.1 | 30 | 289 | 20 | 315 | 20 | Epicatechin |
| **DimerEthylBridge3** | Ethyl Bridged procyanidin dimer | 605 | 26.5 | 30 | 289 | 20 | 315 | 20 | Epicatechin |
| **CafAspAc** | Caffeoyl aspartic acid | 294 | 6.9 | 25 | 88 | 30 | 135 | 30 | Querc-O-glc |
| **Unk632** | Unknown compound at m/z 632 | 632 | 6.4 | 60 | 356 | 35 | 494 | 25 | Dimer A2 |
| **Unk635-1** | Unknown compound at m/z 635 | 635 | 18.7 | 40 | 603 | 20 | 451 | 15 | Dimer A2 |
| **Unk635-2** | Unknown compound at m/z 635 | 635 | 24.1 | 40 | 603 | 20 | 451 | 15 | Dimer A2 |
