# Supplementary material for: Multiblock Analysis to Relate Polyphenol Targeted Mass Spectrometry and Sensory Properties of Chocolates and Cocoa Beans
Source: Metabolites. 2020 Jul 29;10(8):311. doi: 10.3390/metabo10080311 (PMC7465875; doi:10.3390/metabo10080311)
Supplement: Supplementary file 1 [file metabolites-10-00311-s001.zip › supplementary/Submission special issue Metabolites_Supplementary file S2_200527.docx]

**Table S2.** Multiple Reaction Monitoring (MRM) acquisition parameters for chocolate extracts in the negative ion mode.

| **Compound Code** | **Molécule** | | **Precur-sor Ion (m/z)** | | **Reten-tion Time (min)** | | **Cone Volta-ge (V)** | **Qualif. ion (m/z)** | **Coll. Energy (V)** | | **Quantif. ion (m/z)** | | **Coll.  Energy (V)** | | **Quantified as equivalent of** | | |
| --- | --- | --- | --- | --- | --- | --- | --- | --- | --- | --- | --- | --- | --- | --- | --- | --- | --- |
| **Catechin** | Catechin | 289 | | 6.1 | | 55 | | 205 | | 20 | | 245 | | 10 | | Catechin |  |
| **Epicatechin** | Epicatechin | 289 | | 12.3 | | 55 | | 205 | | 20 | | 245 | | 10 | | Epicatechin |  |
| **DimerB1** | Procyanidin dimer B1 | 577 | | 4.7 | | 32 | | 289 | | 20 | | 425 | | 10 | | Dimer B1 |  |
| **DimerB2** | Procyanidin dimer B2 | 577 | | 11.5 | | 32 | | 289 | | 20 | | 425 | | 10 | | Dimer B2 |  |
| **Dimer B4** | Procyanidin dimer B4 | 577 | | 6.2 | | 32 | | 289 | | 20 | | 425 | | 10 | | Dimer B4 |  |
| **DimerB5** | Procyanidin dimer B5 | 577 | | 20.5 | | 32 | | 289 | | 25 | | 425 | | 10 | | Dimer B5 |  |
| **DimerUnk** | Unknown Procyanidin dimer | 577 | | 13.7 | | 32 | | 289 | | 20 | | 425 | | 20 | | Dimer B1 |  |
| **A-type-Dimer** | Procyanidin A-type dimer | 575 | | 14.5 | | 60 | | 423 | | 10 | | 449 | | 20 | | Dimer A2 |  |
| **Trimer1** | Procyanidin trimer | 865 | | 10.3 | | 45 | | 287 | | 30 | | 695 | | 20 | | Trimer C1 |  |
| **Trimer2** | Procyanidin trimer C1 | 865 | | 14.3 | | 45 | | 287 | | 30 | | 695 | | 20 | | Trimer C1 |  |
| **Trimer3** | Procyanidin trimer | 865 | | 15.4 | | 45 | | 287 | | 30 | | 695 | | 20 | | Trimer C1 |  |
| **Trimer4** | Procyanidin trimer | 865 | | 21.4 | | 45 | | 287 | | 30 | | 695 | | 20 | | Trimer C1 |  |
| **TrimerA** | A-type procyanidin trimer | 863 | | 13.5 | | 42 | | 411 | | 40 | | 711 | | 20 | | Dimer A2 |  |
| **Tetramer1** | Procyanidin tetramer | 576^(2-)^ | | 12.6 | | 20 | | 125 | | 30 | | 407 | | 20 | | Catechin |  |
| **Tetramer2** | Procyanidin tetramer | 1153 | | 15 | | 50 | | 407 | | 50 | | 863 | | 20 | | Catechin |  |
| **Tetramer3** | Procyanidin tetramer | 576^(2-)^ | | 22.4 | | 20 | | 125 | | 30 | | 407 | | 20 | | Catechin |  |
| **Pentamer1** | Procyanidin pentamer | 720^(2-)^ | | 14.1 | | 26 | | 125 | | 40 | | 289 | | 20 | | Catechin |  |
| **Pentamer2** | Procyanidin pentamer | 720^(2-)^ | | 15 | | 30 | | 125 | | 40 | | 289 | | 20 | | Catechin |  |
| **Pentamer3** | Procyanidin pentamer | 720^(2-)^ | | 16.4 | | 30 | | 125 | | 40 | | 289 | | 20 | | Catechin |  |
| **Hexamer1** | Procyanidin hexamer | 864^(2-)^ | | 13.3 | | 38 | | 125 | | 50 | | 289 | | 30 | | Catechin |  |
| **Hexamer2** | Procyanidin hexamer | 864^(2-)^ | | 15.9 | | 38 | | 125 | | 50 | | 289 | | 30 | | Catechin |  |
| **Hexamer3** | Procyanidin hexamer | 864^(2-)^ | | 17.8 | | 35 | | 125 | | 50 | | 289 | | 45 | | Catechin |  |
| **Heptamer** | Procyanidin heptamer | 1008^(2-)^ | | 17.4 | | 30 | | 289 | | 25 | | 407 | | 30 | | Catechin |  |
| **DimerB+hexose** | B-type dimer C-hexoside | 739 | | 13.1 | | 40 | | 289 | | 30 | | 329 | | 20 | | Dimer B1 |  |
| **DimerA+hexose** | A-type dimer O-hexoside | 737 | | 19.9 | | 47 | | 449 | | 30 | | 611 | | 20 | | Dimer A2 |  |
| **DimerA+pentose** | A-type dimer O-pentoside | 707 | | 20.3 | | 50 | | 325 | | 50 | | 581 | | 20 | | Dimer A2 |  |
| **Unk404** | Unknown compound at m/z 404 | 404 | | 1.3 | | 22 | | 293 | | 10 | | 337 | | 20 | | Catechin |  |
| **Unk431** | Unknown compound at m/z 431 | 431 | | 11.6 | | 40 | | 266 | | 20 | | 414 | | 20 | | Catechin |  |
| **Unk785** | Unknown compound at m/z 785 | 785 | | 20.5 | | 30 | | 289 | | 25 | | 349 | | 20 | | Catechin |  |
